# Work satisfaction, quality of life, and leisure time of neonatology fellows and senior neonatologists in Israel

**DOI:** 10.1186/2045-4015-1-50

**Published:** 2012-12-14

**Authors:** Michael Moshe, Zvi H Perry, Liat Salzer, Ehud Zemora, Asaf Toker

**Affiliations:** 1 Joyce and Irving Goldman Medical School, Ben-Gurion University of the Negev, Beer Sheva, Israel; 2 Department of Epidemiology and Health Services Evaluation, Ben-Gurion University of the Negev, Beer-Sheva, Israel; 3 Surgical Department A, Soroka University Medical Center, Beer-Sheva, Israel; 4 Department of Health Systems Management, Ben-Gurion University of the Negev, Beer-Sheva, Israel; 5 Central Administration, Hadassah Hebrew University Medical Center, Jerusalem, Israel; 6 Pediatric Division, Soroka University Medical Center, Beer-Sheva, Israel

**Keywords:** Work Satisfaction, Quality of life, Leisure time, Neonatology, Israel

## Abstract

**Objectives:**

To examine work satisfaction, quality of life, and leisure time of neonatology fellows and senior neonatologists in Israel.

**Methods:**

A validated questionnaire was delivered during the second half of 2008 to all the neonatology fellows and senior neonatologists in Israel. Descriptive analysis, parametric Student’s *t*-test, and aparametric Mann Whitney and χ2 tests were conducted.

**Results:**

Of 114 practicing neonatologists in that period in Israel (including both seniors and fellows), 112 (98.25%) participated in the study. The majority of neonatologists were male (53.2%), married (91.7%), 40–60 years old (69.7%), and studied in Israeli medical schools (62.0%). Most did their pediatric residencies and fellowships in Israel (97.2% and 75.7%, respectively). The average number of night/on-call shifts of fellows and senior neonatologists was 8.8 per month (SD ± 3.425) and the number of active on-call shifts was 4.04 (SD ± 3.194). The satisfaction level of neonatologists in Israeli medical centers with patient care, self-reward, work relations, and quality of life was high, but their satisfaction level with workload, income and prestige, and leisure time was low. The general index of work satisfaction and the general index of indices were both high in relation to the mid-range values. The majority of neonatologists stated that they would choose to practice medicine again. Most of them would encourage medical students to choose the same specialty they had chosen. Only a few neonatologists were contemplating changing their choice of specialty. Most neonatologists want to continue practicing medicine; however, a significant number will not recommend that their children do so.

**Conclusions:**

The satisfaction level of neonatologists in Israel is high, mainly due to satisfaction with their work. High satisfaction levels promise high quality patient care, as well as high satisfaction levels of patients and their families. However, satisfaction with leisure time was low and it will require greater attention and focused steps to correct this.

## Background

It has been shown that physicians’ work satisfaction is influenced by factors such as patient care, workload, income and prestige, self-fulfillment, and relations with fellow co-workers [1]. Factors that negatively influence physicians’ work satisfaction [2,3] include time, pressure, lack of leisure time, and time for one’s self, a decline in quality of life, feelings of less autonomy in decision making, less time spent on actual patient care, and more time spent doing administrative work.

No difference in work satisfaction was found [4] among physicians who dedicate most of their time to patient care compared to physicians who dedicate their time to medical research. Nevertheless, physicians who spend more time with patients reported a greater insult to their quality of life. The determining factors for their dissatisfaction were less time for their families, themselves, and leisure activities.

Burnout is a “psychological syndrome of emotional exhaustion, depersonalization and low sense of achievement” [5]. Among physicians, it is a long-term stress reaction that influences work satisfaction and derives from some characterizing factors of today’s medical profession. These factors include physicians’ loss of autonomy and prestige, the financial transformation of health systems [6], work stress, and time pressure, and to some extent from personality traits of physicians. Personality traits such as obsessiveness and rigidity, together with high levels of intelligence, might lead physicians to feel accountable and overcommitted [7].

In the beginning of the burnout process among physicians [5], one can find a group of general background variables such as age, number of children, marital status, and work hours, which interact with mediating variables, namely autonomy at work, home–work relations, and home support. When the background variables combine well with the mediating variables (meaning the physician is practicing in his or her field of choice, is satisfied with work hours, manages to keep a good balance between work and private life, and is getting good support from his or her family), this combination leads to high levels of satisfaction and may prevent burnout. However, when the combination of all the factors is other than perfect, the outcome is most likely to be some degree of burnout.

A sense of burnout is directly related to dissatisfaction, which in turn may lead to a decline in the desire of the physician to treat patients, a tendency to order expensive laboratory examinations, and an ongoing interest in early retirement. Burned-out physicians [7] suffer from social isolation, lack of joy and happiness, depression, and denial. Eventually they become cynical and ironic.

It is likely that the relatively high rates [6] of mental illness among physicians (male and female alike) and the high rates of suicide attempts are related to the combination of problematic work conditions and work dissatisfaction together with some primary predisposing personality factors.

In comparison to male physicians [8], female physicians reported greater levels of dissatisfaction with their autonomy at work and time pressure, and reported 1.6 times greater rates of burnout. Among female physicians who practice surgery [9] there were differences in academic ranking compared to male physicians, as well as differences in level of income, work status, and work relations. The research also showed that almost 30% of the female physicians are seriously considering leaving their work because of these differences.

Despite these findings, a large study [10] of 12,474 physicians in the United States did not find any differences between male and female physicians in terms of work satisfaction.

There is a clear connection [10] between work satisfaction and the type of specialty chosen. Physicians who practice “procedural” fields such as obstetrics and gynecology, ophthalmology, and orthopedic surgery, stated greater dissatisfaction with their work than physicians who practice “non-procedural” fields such as geriatric medicine, pediatric medicine, and dermatology. The difference between the groups was caused by the changes in the health care systems in the United States in the last few years, which affected the social status of physicians, their autonomy, and their salaries.

In the survey conducted by Clarke and Associates, which focused on job satisfaction and stress among neonatologists, it was found that almost all neonatologists experienced stress at work: 34% moderately severe and 16% very severe stress. Major causes of stress were excessive work load, on call too often, and calls at night; problems in patient care, especially dealing with infant death; and staff disagreements, especially nurse or house staff conflicts. One-sixth of the neonatologists were either moderately or very dissatisfied with their career. Major dissatisfactions were: too much work, especially managing many sick patients; lack of resources, including inadequate salary; too much stress at work; and administrative demands. Job satisfaction was derived from patient care, teaching, intellectual stimulation, and research. Altering their subspecialty had been considered at some time by 58% (15% very seriously). The researchers also suggested that job stress is a greater problem than job dissatisfaction [11].

Overall, the satisfaction level of physicians in the last few decades has been declining [12-14]. Since the 1970s, work dissatisfaction among physicians doubled from less than 15% to more than 30% in the late 1990s. About 40% of the physicians stated they would not have chosen to practice medicine again if given the chance, and a larger percentage said they will not recommend their children do so. [15] In addition, 41% of the physicians have contemplated leaving the medical profession because of their discontent [16].

Similar to the above mentioned changes, the choice of medical specialty by medical students in the United States went through great changes in the last few years [17]. A study conducted between 1996 and 2002 found that specialty fields such as dermatology and anesthesiology, which traditionally had very low rates of interest among the graduating students, had multiplied the number of their candidates by tens and hundreds of percentages (anesthesiology – a rise of 500%, dermatology – a rise of 1000%). More than 55% of the students said that the factor that most strongly influenced their choice was the ability to have a “controllable lifestyle”, meaning having time that is free of the demands of their work, that could be dedicated to their leisure activities and families, and which is controlled by them. A similar change was noticed among Israeli medical school graduates [18], which demonstrated a rise in the popularity of specialties such as radiology and anesthesiology between 1980 and 1995. Unfortunately, it is not the case in neonatology in Israel – the number of physicians choosing neonatology as a subspecialty is constantly insufficient, an average of 5 per year in the last 20 years [19], whereas the number of live births is steadily increasing [20]. The severity of situation, caused the Amourai Committee in 2002 to declare neonatology as one of the “in crisis specialties” [21]. In contrast, in the United States, the number of neonatologists rose by 150%, whereas the number of premature babies had risen only by 18% [22].

The current study aimed to describe the level of work satisfaction, quality of life, and leisure time of senior neonatologists and neonatologist fellows in Israel by age, gender, status in their department, and time in the profession. The term “neonatology fellowship” is being used here to refer to advanced training in neonatology. All participants in the neonatology training programs have already completed a basic residency in pediatrics. In Israel, pediatricians participating in such advanced training are often referred to as “residents in neonatology”, with the training in neonatology functioning as a second (advanced) residency.

We asked all active senior neonatologists and neonatologist fellows in Israel, between July 2008 and March 2009, to participate in the current study. In Israel there are 25 medical centers specializing in neonatal care, with a total of 114 practicing neonatologists. Most of these centers provide neonatal services including prenatal counseling for high risk pregnancies, neonatal care in the delivery room, normal postnatal follow-up and care, services for neonates with congenital defects with follow-up, treatment in special care units and in neonatal intensive care units (NICU), and neonatal counseling. In Israel there were 151,679 live-births in 2007 [20]. This number is consistent with a steady rise in live births over the last 50 years. At the same year, according to the Ministry of Health data, the number of beds for special neonatal care in Israel was only 556 [23]. In comparison, according to a workforce report from the American Academy of Pediatrics in October 1996, there were at that time 3688 board-certified and board-eligible neonatologists in the United States. This means that in the U.S. there are 1.2 neonatologists per 10,000 people in comparison to 1.4 neonatologists per 10,000 people in Israel. This seems a fair comparison until we compare the live birth ratio, which is 14/1000 in the US vs. 20/1000 in Israel.

## Methods

### Type of study

Cross-sectional research that examined basic feelings and positions among all senior physicians and fellows in neonatology in Israel.

### Study population

In Israel there are 118 neonatologists of working age. Of these, 114 were active and practicing in Israel at the time the survey was conducted; 112 (98.25%) participated in the current research. A neonatologist is defined as a physician who possesses an Israel Ministry of Health certificate in neonatology or one that has begun his neonatology fellowship, but did not yet finish it.

### Variables

Dependent variables: satisfaction level, quality of life, and leisure time.

Independent variables: age, gender, place of medical education, and country of origin.

### Tools

Research questionnaires: Based on similar questionnaires [1,4,14,24], a questionnaire was designed by the researchers to include questions about work satisfaction and quality of life. Additional questions about leisure time were composed by the researchers, based on prior studies [25]. The work satisfaction section of the questionnaire was divided into five sub-categories: Patient care, Work load, Income–prestige, Self–reward, and Work relations. The questionnaire used a seven-point Likert scale (1 = totally dissatisfied, 7 = very satisfied). Each category was summed to create an index measurement. The sum of these five indices was used to create a summative index of work satisfaction. Different indices were created for the questions about the quality of life and leisure time. Finally, in order to demonstrate the relation between work satisfaction, quality of life, and leisure time, we created the index of indices that summarizes all the questions regarding these issues.

The next stage was the actual mail/fax/hand delivery of the questionnaires to the neonatologists in the hospitals, after receiving their permission. A researcher was available to answer any questions, and received the questionnaires in the same way they were sent. The participants were all promised full confidentiality.

### Statistical analysis

The data were coded into the SPSS 13 computer program for Windows (Chicago, IL). In order to make sure there were no errors in entering data, we explored frequency checks for all the variables. Questionable parameters were re-checked with the original questionnaire, and in cases where no decision could be made the values were considered missing. The data were analyzed using descriptive statistics first, and then with parametric Student’s *t*-test and non-parametric Mann–Whitney and χ2 tests. P value less than 0.05 was considered significant.

In order to measure the level of satisfaction, we compared the relevant central measurement with the mid-range value, so that when the central measurement (median or mean value) was higher than the mid-range value the satisfaction was considered high.

## Results

### Demographic data (Table 1)

**Table 1:** Demographic data of neonatologists in Israel (N = 112)*

**Demographic variable**	**Number of responders (n)**		**Percentage**
Gender	Male	58	53.2
	Female	51	46.8
Age (years)	31-40	18	16.5
	41-50	43	39.4
	51-60	33	30.3
	61-65	15	13.8
Familial status	Single	3	2.8
	Married	99	91.7
	Divorced	6	5.5
Status on ward	Ward director	19	17.4
	Unit director	21	19.3
	Senior	50	45.9
	Fellow	19	17.4
Country of birth	France	2	1.8
	Israel	59	54.6
	Other	22	20.4
	Romania	7	6.5
	USA	3	2.8
	USSR	15	13.9
Country of neonatology fellowship	Canada	1	0.9
	Israel	69	64.5
	Israel & Canada	2	1.9
	Israel & Other	13	12.1
	Israel, S. Africa & USA	6	5.6
	Israel & USA	4	3.7
	S. Africa	4	3.7
	USA	8	7.5

* Not every question was answered by all participants.

In Israel there were 118 senior physicians and fellows in neonatology of working age at the time of the survey. All of them were qualified specialists in pediatrics. Of those, 114 were practicing neonatologists, and 112 (98.25%) participated in the current study. The majority of neonatologists were male (53.2%), married (91.7%), 40–60 years old (69.7%), and studied in Israeli medical schools (62.0%). Most of them did their pediatric residencies and fellowships in Israel (97.2% and 75.7%, respectively). The average number of night/on-call shifts of fellows and senior neonatologists was 8.8 per month (SD ± 3.425) and the number of active on-call shifts (when the senior physician had to attend the hospital) was 4.04 (SD ± 3.194).

### Satisfaction level (Tables 2 and 3)

**Table 2:** Satisfaction level measurements of the neonatologists in Israeli medical centers (N = 112)

**Number**	Variable*	**Range of values**	Mid-range value**	**Neonatologists’ Average (S.D.)**	**Residents’ Average (S.D.)**	***p *value**
**1**	**Index of patient care**	19-56	37.5	39.89 (± 7.38)	37.3 (±7.7)	0.001
**2**	**Index of workload**	8-40	24	22.36 (±6.96)	18.85 (±7.53)	<0.001
**3**	**Index of income/prestige**	8-44	26	23.84 (±7.87)	26.14 (±10)	0.049
**4**	**Index of self reward**	9-42	25.5	31.02 (±6.07)	29.92 (±6.63)	0.18
**5**	**Index of work relations**	11-28	19.5	22.99 (±3.39)	22.46 (±3.45)	0.22
**6**	**General index of work satisfaction**	83-189	136	140.28 (±22.37)	132.85 (±10.84)	<0.001
**7**	**Index of quality of life**	26-78	52	60.59 (±9.17)	55.06 (±12.56)	<0.001
**8**	**Index of leisure time**	10-70	40	39.42 (±12.52)	27.7 (±28.5)	<0.001
**9**	**General index of all indexes**	131-319	225	240.17 (±33.21)	216.7 (±41.3)	<0.001

* Every index is the sum of points for all questions in that specific section of the questionnaire.

** Satisfaction was considered high when the average value is greater than the mid-range value and low when the average value is less than the mid-range value.

**Table 3:** Satisfaction level measurements of the neonatologists in Israeli medical centers in comparison to mid-range values (N = 112)

**Number**	Variable*	**Range of values**	Mid-range value**	**Neonatologists’ Average (S.D.)**	**p value (single sample t-test)**
**1**	**Index of patient care**	19-56	37.5	39.89 (± 7.38)	<0.001
**2**	**Index of workload**	8-40	24	22.36 (± 6.96)	0.014
**3**	**Index of income/prestige**	8-44	26	23.84 (± 7.87)	0.004
**4**	**Index of self reward**	9-42	25.5	31.02 (± 6.07)	<0.001
**5**	**Index of work relations**	11-28	19.5	22.99 (± 3.39)	<0.001
**6**	**General index of work satisfaction**	83-189	136	140.28 (± 22.37)	0.046
**7**	**Index of quality of life**	26-78	52	60.59 (± 9.17)	<0.001
**8**	**Index of leisure time**	10-70	40	39.42 (± 12.52)	0.63
**9**	**General index of all indexes**	131-319	225	240.17 (± 33.21)	<0.001

The satisfaction level of neonatologists in Israeli medical centers with patient care, self-reward, work relations, and quality of life was high, but their satisfaction level with workload, income and prestige, and leisure time was low in relation to the mid-range value. As a result, the general index of work satisfaction and the general index of indices were both high in relation to the mid-range values.

### Leisure time (Table 4)

**Table 4:** Satisfaction of neonatologists at Israeli medical centers with the frequency of leisure time activities (N = 112)

**Number**	**Leisure activity**	Frequency of neonatologists*	**Frequency of residents**	**p value**	Median of satisfaction**
**1**	**Meeting family**	2.85 ± 2.21 (per month)	1.35 ± 2.01	<0.001	5
**2**	**Restaurants**	10.86 ± 10.56 (per year)	1.35 ± 1.69	<0.001	5
**3**	**Sports**	9.02 ± 9.04 (per month)	1.5 ± 1.27	<0.001	4
**4**	**Trips**	4.67 ± 5.28 (per year)	2.2 ± 1.8	<0.001	4
**5**	**Hobbies**	4.95 ± 8.05 (per month)	1.4 ± 1.44	<0.001	4
**6**	**Vacation abroad**	1.34 ± 1.11 (per year)	1.3 ± 0.68	0.72	4
**7**	**Book reading**	1.18 ± 3.42 (per month)	4.4 ± 2.5	<0.001	4
**8**	**Meeting friends**	3.21 ± 2.76 (per month)	1.16 ± 1.85	<0.001	4
**9**	**Vacation in Israel**	1.28 ± 1.36 (per year)	1.5 ± 1.12	0.17	3
**10**	**Movies**	4.28 ± 7.55 (per year)	1.4 ± 0.73	<0.001	3

* Number of times the activity took place (and standard deviation) according to the time frame indicated.

** Satisfaction level was measured on a scale of 1–7. Satisfaction is considered high when the median value is greater than the mid-range value (3.5), and is considered low when the median value is less than the mid-range value.

The satisfaction level of the neonatologists in Israeli medical centers when looking at their leisure time was generally higher than the mid-range value. They were not satisfied with the frequency with which they went to the movies and the number of vacations in their home country, but were satisfied with all other mentioned activities.

### Neonatologists’ opinions about the medical profession

The majority of neonatologists (n = 98, 87.5%) stated that, given the chance, they would again have chosen to practice medicine. Most of them (n = 78, 69.6%) would encourage medical students to choose the same specialty they had chosen. Only a few (n = 7, 6.25%) neonatologists were contemplating changing their choice of specialty. As for leaving the medical profession, most neonatologists (n = 99, 88.4%) want to continue practicing medicine; however, a high number (n = 39, 34.8%) of the neonatologists in Israel will not recommend that their children practice medicine.

We compared the satisfaction level between different groups of neonatologists:

A) Senior neonatologists, compared with fellows after the pediatric residency alone, were more satisfied regarding personal satisfaction (*p* = 0.009) and more satisfied with their quality of life (*p* = 0.042).

B) Regarding job benefits, neonatologists who got their medical education in Israel were less satisfied than neonatologists who studied medicine outside of Israel (*p* = 0.008). We failed to detect any difference between male and female fellows regarding quality of life and leisure time.

We also compared the satisfaction level between neonatologists and fellows (as described in our previous research [25]). The results in Table 2 show that neonatologists have higher indices such as patient care, workload, income, work satisfaction, leisure time, and the general index than fellows.

## Discussion

It is a known fact that work satisfaction of physicians’ worldwide has been declining in the last few decades. Important reasons for this decline in satisfaction include lower income levels, reduced autonomy and social status, a change in perception about the physician’s place in society, lack of self-fulfillment, enormous time pressure at work, and lack of time for one’s family and self. Research has shown that dissatisfaction of physicians leads to burnout, causes ongoing desire to leave the medical profession, and eventually causes dissatisfaction with their patients [26,27].

The current study is the first report from Israel providing reliable data about work satisfaction, quality of life, and leisure time of neonatologists. It shows that the overall work satisfaction and general satisfaction levels are high, and the reason for this is likely due to a strong sense of self-reward and satisfaction with their quality of life.

### Work satisfaction

Work satisfaction level alone, apart from leisure time, among the neonatologists was high, especially with patient care, sense of self-reward, and work relations. High satisfaction levels are well-matched with our findings that a majority of the neonatologists surveyed would choose to practice medicine again, would recommend that medical students choose their line of specialty, and are not considering a change of internship or contemplating a change in profession. But the satisfaction levels from workload and income were low. These low satisfaction levels matched a significant number of neonatologists who stated that they would not recommend that their children practice medicine. Moreover, it is reasonable to presume that these low satisfaction levels have direct implications on neonatology as a profession in crisis in Israel, and upon the unwillingness of young physicians to choose neonatology as their subspecialty. Dissatisfaction with workload and income level correlates well with similar findings from the United States [2], where 600 physicians showed a substantial decline in their satisfaction level, especially due to increasing workload (which leaves less time for each patient) and the lack of financial reward for the work being done. Another study [3] points out that the balance between factors that positively influence satisfaction level (such as patient care) and factors that have a negative influence on satisfaction levels (such as workload and the lack of time for patients and for one’s self), must be vigorously kept. When this balance fails to be maintained, burnout and dissatisfaction prevail.

### Quality of life and leisure time

Despite the high levels of work satisfaction found among neonatologists at Israeli medical centers, we found low levels of satisfaction with their leisure time. Surprisingly, the reported quality of life was high, in opposition to what we found in a recent survey of fellows from one Israeli medical center [25].

These findings are similar to physicians’ [2] feelings that their work prevents their having leisure time and emphasizes the importance of leisure time in maintaining a good quality of life. Engaging in leisure time activities, such as spending time with one’s family and friends, and being engaged in physical activities, can prevent burnout. The most crucial factors that influence physicians’ work satisfaction [4] were their quality of life, time spent with their families, and personal time for themselves and for leisure time. These factors have created a change in the specialty choices of graduating medical students [17] in the United States in the last few years. Specialty fields that are perceived as less time intensive are gaining popularity. This means that the issues of quality of life and leisure time are becoming more and more important as determining factors of physicians’ satisfaction.

### Female vs. male neonatologists

Satisfaction levels were similar among female and male neonatologists. Some studies support our results [1,10]. However, among 5704 U.S. physicians [8] differences were found between male and female physicians. Women were more satisfied than men with their work relations and field of choice, but were less satisfied with their autonomy at work, workload, and salary. Differences between genders [9] were also based on salary issues, prestige, and academic ranking. The fact that we did not find any difference between male and female physicians is intriguing, since we would expect that in at least one parameter – academic ranking – women would be less satisfied than men in Israel, the same as abroad, because they do not climb as fast as men in academic ranking [28].

### Neonatology fellows vs. senior neonatologists

Satisfaction levels were generally similar between physicians in the course of fellowships and senior neonatologists. Some studies [29] show an inverse relation between the fellow’s age and level of work satisfaction. The highest satisfaction level was demonstrated among very young and very old physicians [10], yet satisfaction level was directly related to age and experience [30].

In our study we found, in general, that fellows were as satisfied as seniors. The only exceptions were found among neonatology fellows with lower personal satisfaction and quality of life. It is logical to find these results, as it is well known that fellows have more on-call shifts and need to work harder due to their relative lack of experience in the field. These findings add to the results from other studies [1-3,8-10,13] that emphasize the importance of income level as a factor in determining work satisfaction.

Possible explanations for the nearly similar findings between these two groups may be the fact that fellows in neonatology have already completed their pediatric residencies and the length of their neonatology fellowship is shorter.

### Neonatologists from Israeli medical schools vs. neonatologists from non-Israeli medical schools

The place of medical education further explains the differences found between the two groups – Israeli fellows were less satisfied with issues regarding personal benefits from the job, as well as their workload. The low levels of satisfaction with workload among Israeli graduates is similar to that reported in another study [31] conducted in Israel, which found that the satisfaction level of immigrant physicians was higher regarding workload and burnout levels. Regarding self-reward, it is possible that the long and difficult course immigrant physicians must go through in order to work in Israel, combined with the difficulties of immigrating to another country, had a less negative influence on their sense of self-reward after becoming senior neonatologists, especially in comparison to Israeli graduates [32,33]. Another finding that may explain the low satisfaction levels of Israeli graduates is that many more Israeli medical school-trained neonatologists have an additional academic degree, which means they spent more years in the university and thus have higher expectations.

The low levels of satisfaction among Israeli graduates found in the study are worrisome since it is well-established that low levels of satisfaction can eventually lead to burnout [5]. This burnout might have important personal implications for physicians as well as serious consequences for the health system at large. Burned-out physicians tend to be more angry and impatient, and tend to develop signs of depression, substance abuse, and suicide [34]. Burned-out physicians also report greater levels of exhaustion [35], which may lead to medical errors, impair their ability to learn and concentrate, and damage their professional and personal relations. From a wider perspective [5], burnout causes poor doctor–patient relations and a decline in the quality of care, and thus surely needs to be addressed.

### Neonatologists vs. fellows

Neonatologists were found to have higher indices in patient care, workload, income, work satisfaction, leisure time and the general index than fellows. But, this is a very problematic comparison – they compared to fellows, who are the main working force and most battered physicians in the field, and thus this improvement is nothing to brag about. Even worse is the fact that two important indices were not better with neonatologists – the index of self-reward and the index of work relations. This means that at the end of the day senior physicians, our neonatologists, did not fare better than simple fellows in the main issues that cause burn-out – self-fulfillment and work relations, things that should cause us to wonder how this may impact their motivation and working ability.

### Limitations of the current study

The questionnaires were sent by mail, fax, or distributed by hand, and filled out by neonatologists on their own time. Therefore, the opportunity to ask questions was only possible by contacting the researchers by telephone.

The response rate in this study was about 98.3%; however, not all the questions in all the questionnaires were answered.

## Conclusion

The satisfaction level of neonatologists in Israeli medical centers is high, mainly due to high satisfaction with their work and to a lower extent to their quality of life. High satisfaction levels promise high quality patient care, as well as high satisfaction levels of patients and their families. However, satisfaction with leisure time was low and it will require greater attention and focused steps so that these issues can be corrected. We recommend that further research be conducted, using the tools developed in our study, that will compare the results in neonatology with physicians in other areas of medicine in Israel and worldwide. Ultimately, a comprehensive picture about the satisfaction level with leisure time in Israel and the world can be created, and ways found to improve it. We also recommend a change in management policies be made in order to resolve these problems.

## Competing interests

The authors declare that they have no competing interests.

## Authors’ contributions

ZHP analyzed the data and drafted the paper. AT& EZ directed were responsible for study design and edited the paper. MM and LS were responsible for data collection. All authors read and approved the final manuscript.

## Authors’ information

Michael Moshe is a resident in plastic surgery, Beilinson Medical Center.

Zvi H. Perry is a resident in Surgical Department A, Soroka University Medical Center, Beer-Sheva, Israel, and a PhD student in the Department of Epidemiology and Health Services Evaluation, Ben-Gurion University of the Negev, Beer Sheva, Israel.

Liat Salzer is a resident in gynecology, Tel-Hashomer Medical Center.

Ehud Zmora is head of the Neonatology Ward, Soroka University Medical Center.

Asaf Toker is vice-president, Hadassah Hebrew University Medical Center, Central Administration, Jerusalem, Israel.
